# Bio-Inspired Band-Gap Tunable Elastic Optical Multilayer Fibers

**DOI:** 10.1002/adma.201203529

**Published:** 2013-01-27

**Authors:** Mathias Kolle, Alfred Lethbridge, Moritz Kreysing, Jeremy J Baumberg, Joanna Aizenberg, Peter Vukusic

**Affiliations:** Harvard University, School of Engineering and Applied Sciences9 Oxford Street, Cambridge, MA-02138, USA E-mail: mkolle@seas.harvard.edu; University of Exeter, School of PhysicsStocker Road, Exeter, EX4 4QL, UK E-mail: P.Vukusic@exeter.ac.uk; L. Maximilians University, Systems Biophysics, Department of PhysicsAmalienstr. 54, München, D-80799, Germany; University of Cambridge, Nanophotonics Centre, Cavendish LaboratoryJJ Thompson Ave, Cambridge, CB3 0HE, UK

Knowledge of the interplay between the morphology, composition and optical appearance of biological photonic systems can provide broad inspiration for novel artificial photonic elements.^1^–^3^ On occasion, the study of natural photonics yields specific design templates for optical technologies.^4^–^9^ To this end, we present the results of the investigation of the hierarchical photonic structure discovered in the seed coat of *Margaritaria nobilis* fruits, which directly inspired our creation of novel photonic fibers. The fruit's hue results from the interference of light within a concentrically-layered architecture found inside individual cells in the seed's outer tissue layers. The natural structure presents two codependent, technologically exploitable features for light and color manipulation: regularity on the nanoscale that is superposed with microscale cylindrical symmetry, resulting in wavelength selective scattering of light in a wide range of directions. This is the foundation for novel soft bio-inspired photonic fibers with the spectral filtering capabilities and color brilliance of a planar Bragg stack compounded with a large angular scattering range introduced by the microscale curvature, which also decreases the strong directional chromaticity variation usually associated with flat multilayer reflectors. Transparent and elastic synthetic materials equip the multilayer interference fibers with high reflectance that is dynamically tuned by longitudinal mechanical strain. A two-fold elongation of the elastic fibers results in a shift of reflection peak center wavelength of over 200 nm. The bio-inspired design and manufacture of this form of soft photonic fiber heralds the transition to novel fiber-based flexible photonic materials and textiles with colors that are tunable over the entire visible spectrum and optical strain sensors.

Nature's most vivid colors, highest transparencies, strongest whites and deepest blacks rely on ordered, quasi-ordered or disordered structures with lattice constants or scattering element sizes on the order of the wavelength of visible radiation.^10^–^16^ By inducing interference or diffraction, biological photonic structures of a wide structural diversity strongly alter the spectral composition of reflected and transmitted light resulting in the stunning structural colors of many organisms.^17^, ^18^ One-dimensional multilayer arrangements play an important role in the creation of structural colors in nature and have primarily been studied in the animal kingdom, especially the insect world.^10^, ^19^, ^20^ Planar layered photonic system have recently also been increasingly frequently reported in various plants.^21^–^24^

The fruits of the plant *Margaritaria nobilis* in the rain forests of Middle and South America have a striking blue - green hue (**Figure** **1**a). The plant partly relies on seed dispersal by birds which might be attracted by the colorful display.^25^, ^26^ The cells in the fruit's blue seed coat are elongated and mostly appear blue or green (Figure 1a,b). Several layers of cells are stacked on top of each other with varying planar orientation of the individual cell layers (Figure 1c). A single cell cross-section reveals that the entire interior volume is occupied by a periodic concentrically-layered morphology with an overall periodicity of (180 ± 30) nm (Figure 1d, e). Light incident on the fruit's surface undergoes interference within the periodic structure in each cell resulting in the reflection of blue light.

**Figure 1:**
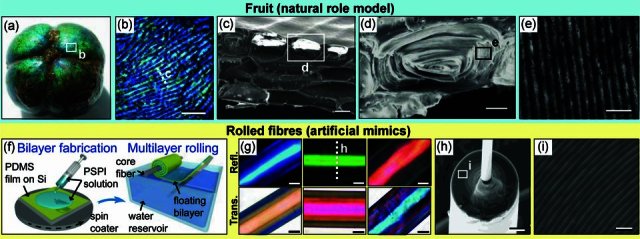
Fruit and fibers. (a) A *Margaritaria nobilis* fruit without its capsule (∼10 mm in diameter). (b) Optical micrograph of the fruit's surface showing the elongated blue cells. (c) A scanning electron micrograph of a cross-section through the outer layers of the fruit's endocarp is showing several stacks of cells. (d) A cross-section through a single tissue cell reveals the interior architecture - a concentric, flattened cylindrical layered structure. (e) A section of the layered architecture within a single cell visualized by transmission electron microscopy. (f) Schematic representation of the manufacturing of artificial photonic fibers. (g) Optical micrographs of three rolled-up multilayer fibers with different layer thicknesses and colors in reflection (top) and transmission (bottom). (h) Scanning electron micrograph of a fiber cross-section showing the multilayer cladding wrapped around the fiber's glass core. (i) Scanning electron micrograph of the individual layers in the cladding. Scale bars: 200 μm (b), 20 μm (c), 10 μm (d), 500 nm (e), 20 μm (g), 20 μm (h) and 1 μm (i).

Under directional illumination a planar multilayer interference structure can only display its bright coloration in the specular reflection direction. The hue of such planar Bragg stacks strongly depends on the angle of incidence. Under diffuse illumination the observed color blue-shifts for increasing observation angle. By contrast, in *M. nobilis* fruits the superposition of a microscopic curvature on the nanoscale regularity of the layered structure within each individual seed coat tissue cell combined with the fruit's overall macroscopic curvature leads to an increased visibility of the reflected structural color across a wide angular range. Under directional illumination, a part of the curved multilayer in a majority of the individual cells is oriented to satisfy the specular reflection condition providing a spatially varying pixelated sparkle of different hues (Figure 1a) that depend on the locally varying angle of light incidence. In diffuse light this sparkle is suppressed as the light reflected by each fruit cell towards the observer originates from light incident on the fruit from various directions, producing a more isotropic color that only gradually changes across the fruit due to the macroscopic curvature.

The emergence of unique structural and optical properties from combinations of structures on different length scales within hierarchical synergistic assemblies is a principle often encountered when studying natural systems.^19^, ^27^ Increasingly frequently this concept is also applied in novel optical technologies.^6^, ^7^, ^9^ The hierarchical photonic architecture in the seed coat of the *M. nobilis* fruit is the key element involved in the creation of intense blue and green hues. It provides inspiration for the manufacture of artificial photonic fibers with the optical functionality being defined by the interplay of nanoscopic regularity and superimposed microscopic curvature. The artificial bio-inspired system presented in this article consists of concentric multilayer-wrapped fibers with a radially periodic refractive index profile built from only two alternating phases. While similar to its natural model in dimensions and underlying optical interactions, the artificial system avoids many of the complexities in the natural structure including the ellipticity of the fruit cells' cross-section and any existing fine structure within the periodic layers.

Optical fiber systems with multilayer claddings have theoretically been discussed since the late 70's and have more recently been manufactured using standard fiber drawing processes from macroscopic preforms.^28^–^32^ The choice of materials that can be drawn into multilayer fibers is constrained to a limited, albeit continuously expanding set of components.^33^ In particular, the preform material combinations need to provide an appropriate refractive index contrast and have matching thermal expansion coefficients in order to prevent fracturing at the material interfaces during the processing at elevated temperature.^33^

Here, we present an alternative approach that allows fabrication of fibers at room temperature from a wide range of soft organic and also inorganic materials with varying optical and mechanical properties that are not restricted to a translational symmetry along the fiber axis as in thermally drawn fibers. The fibers reported in this article consist of two elastomeric dielectrics, polydimethylsiloxane (PDMS) and polyisoprene-polystyrene triblock copolymer (PSPI), two inexpensive materials that are commercially available in industrial quantities and provide a sufficiently high refractive index contrast (*n*_PDMS_ = 1.41 ± 0.02, *n*_PSPI_ = 1.54 ± 0.02, determined by ellipsometry). Multilayer fibers are produced by initially forming a bilayer of the two constituent materials, which is subsequently rolled up onto a thin glass fiber (≍10–20 μm diameter) to form the multilayer cladding (Figure 1f and Figure S1 in the Supporting Information).^34^, ^35^

This material system, which was reported earlier in the context of planar flexible multilayer systems,^34^ was chosen here for its advantageous set of properties for the controlled tuning of the fiber's optical performance, which is demonstrated below. The fiber rolling technique used to produce the fibers has previously been employed for the creation of multilayer claddings on rods with macroscopic diameter to facilitate the manufacture of microscopically planar multilayer stacks. Here, we show for the first time that this technique can be employed to form concentric multilayers on core fibers of only ∼15 μm diameter using elastic materials. Such multilayer fibers with curvature on the microscale display optical properties that are distinctly different from the macroscopic rolls previously produced.^35^

Other dielectric materials that have been used in the fiber manufacture include thermoplastics, for instance polystyrene and poly(methyl methacrylate). Thin metal films have been incorporated in non-stretchable fibers with macroscale diameter employing the rolling technique.^35^ Spray coating or blade coating, two industrially well-established techniques that are compatible with roll-to-roll processing, could be explored as viable alternatives for the bilayer production on a larger scale.

The individual thicknesses of the two films in the initial bilayer can be tuned during the film deposition. Consequently, the spectral position of the reflection band of the fibers can be freely adjusted. Three fibers with high reflectivity in different color ranges and the corresponding complementary color in transmission are shown in Figure 1g. Scanning electron micrograph images of the cross-section of a green fiber visualize the concentric multilayer cladding with 80 periods wrapped around the core glass fiber (Figure 1h, i).

Similar to the optical signature of the tropical fruit (**Figure** **2**a,b), these fibers show a pronounced reflection in a finite wavelength range imposed by the multilayer periodicity (Figure 2d,e) and a corresponding drop in transmission (Figure 2f,g). Fibers rolled with multilayer claddings of up to 150 periods provide a reflectivity of more than 90% in their reflection band and a bandwidth varying from 70 nm to 30 nm, decreasing with increasing number of layers in the cladding.

**Figure 2:**
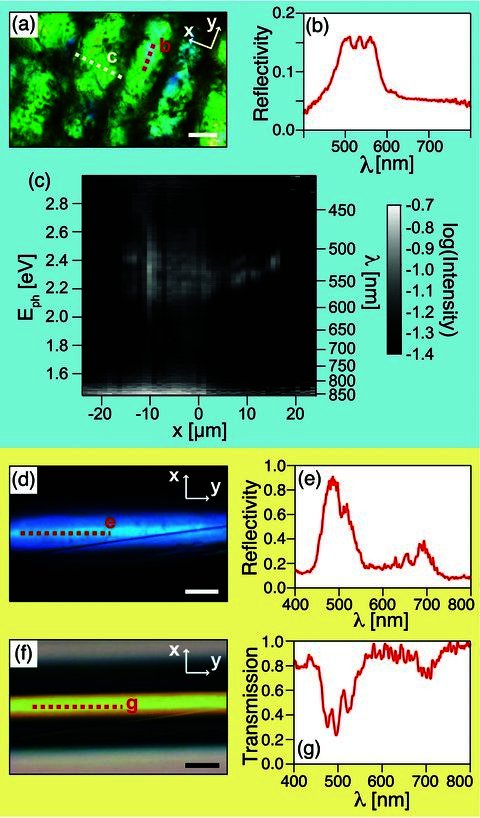
Optical characterization of the fruit and the fibers. (a) Optical micrograph of a fruit's surface visualizing parts of elongated tissue cells. (b) The corresponding reflection spectrum averaged along the red dashed line in (a). (c) Spectrally resolved intensity distribution acquired from a line scan (white dashed line in (a)) radially across a single cell showing the variation of the reflection band induced by the curvature of the layered nanoscale architecture. *E*_ph_ - photon energy, x - spatial position radially along the fiber, centered at the fiber axis. (d) Optical micrograph of a blue fiber in reflection. (e) The corresponding reflection spectrum averaged along the red dashed line in (c). (f) Optical image of the same fiber in transmission. (g) The corresponding transmission spectrum averaged along the red dashed line in (e). All scale bars: 20 μm.

Collection of spectra along a line perpendicular to the fruit cell axis (white dashed line in Figure 2a) allows for the reconstruction of the axially symmetric spatial reflection intensity distribution (Figure 2c). This measurement was also performed on the fibers to determine their reflection and transmission intensity distribution. Note that due to its curvature, the concentric multilayer reflector translates the lateral distance from the fiber's symmetry axis at which each spectrum was taken into a corresponding incident angle (**Figure** **3**a, b). This allows for the direct measurement of the dispersion relation of the multilayer fiber system in reflection and transmission (Figure 3c,d, for details see Figure S2 and discussion in the Supporting Information).

**Figure 3:**
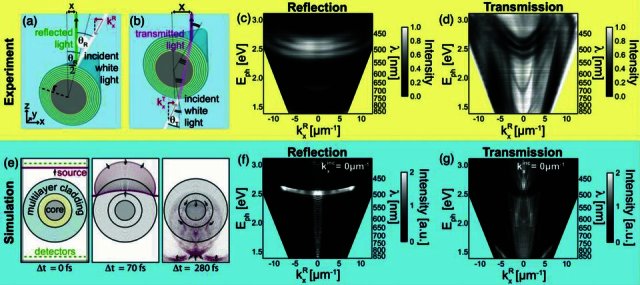
Comparison of optical modeling with experimental data. (a) Simplified schematic representation of light reflection from the fiber into the vertical direction, revealing the relation between projected radial distance *x* from the multilayer fiber symmetry axis and the angle of light incidence *θ*_R_ (effects induced by the finite numerical aperture of illumination and collection optics, including extended light cones have been neglected in this sketch). (b) A similar schematic for light transversely transmitted by the fiber. (c) Band diagram of the fiber reflection for light in the visible range. (d) Band diagram of the fiber transmission. (e) The left image visualizes the FDTD simulation cell set-up used for optical modeling, including the cross-section of the fiber with an inner cladding periodicity of 710 nm (yellow) and an outer periodicity of 340 nm (light blue), the incident light pulse spanning the whole visible range (red and blue), the reflection and transmission detectors (green dashed lines) and absorbing boundaries at the cell edges (black). The following two images show the light pulse propagating transversely through the multilayer fiber at two later points in time and for clarity, the fiber is shown in its contours. In all three images the logarithm of the light pulse's field amplitudes is shown increasing in value from blue to red. (f, g) Reflection and transmission band diagrams deduced from the simulations for perpendicular light incidence (*k*_x_ = 0 m^−1^).

Under directional illumination the difference in radial variation of the peak wavelength from fiber and individual fruit cells results from a subtle difference in geometry. Cross-sections of the fruit cells show that the multilayer is arranged in concentric ellipses aligned with the major axis in the plane of the fruit's surface which leads to a less pronounced color variation across a single cell. In contrast, the multilayer cladding of the artificial fibers has a circular concentric cross-section inducing a radial color variation with larger gradient.

Finite difference time domain (FDTD) simulations of the fibers' optical performance were carried out on a high performance computing cluster using the MIT electromagnetic equation propagation software package (MEEP)^36^ to explain the complexity of the measured dispersion diagrams and to predict the change in optical properties due to a variation in the fiber design. This approach was motivated by the recent investigation of a photonic structure in a fish retina, where FDTD simulations of the interaction of light with the complex hierarchical biological photonic system could provide valuable insight into the interplay of all components on the different length scales.^37^ The simulations presented here result from interactions of light with a fiber of 60 layers with 340 nm periodicity in the outer multilayer cladding zone matching the geometry of the blue fiber from which the experimental dispersion diagrams were obtained. The inner cladding zone adjacent to the fiber core was occupied by a small number of bilayers of ≍710 nm periodicity also found in the real fiber. In the simulations the spatiotemporal electromagnetic field amplitude distributions of light reflected and transmitted by the fiber are collected in the fiber's near-field after excitation with a broadband pulse. An appropriate far-field transformation yields the reflected intensities as a function of frequency or photon energy and wave vector component *k*_x_ - i.e. the dispersion relation of the multilayer fibres.^38^, ^39^ The band structures obtained in these simulations (Figure 3f, g) match the experimentally acquired dispersion diagrams of the fibers (Figure 3c,d). The main Bragg reflection peak at a photon energy of 2.5 eV (corresponding to a wavelength of 496 nm) and its spectral variation with propagation direction is captured in both sets of data for transmission and reflection. A blue-shift of the reflection band is observed with increasing propagation angle in experiment and simulation. However, this spectral variation amounts to less than 40% of the blue-shift expected from a comparable flat multilayer structure, showing that the curvature of the multilayer cladding more than halves the angular variation in chromaticity usually associated with multilayer reflectors. The same effect arises for reflection of light from the multilayered cells in the seed coat of the *M. nobilis* fruit (Figure 3c) explaining its isotropic coloration in diffuse illumination.

In this particular fiber sample, the splitting in the reflection band observed in experiment and simulation is caused by interference of light in the inner zone of the multilayer cladding with ≍710 nm periodicity surrounding the fiber core. High frequency intensity fluctuations as a function of photon energy in the simulations are caused by the fiber core, which acts as a larger resonant cavity introducing an abundance of defect modes. This is not captured in the experimental data due to a limited spectral resolution. In fiber designs with smaller cores these cavity modes would be detectable and potentially even be manifested in angle-dependent spectrally varying intensity fluctuations visible to the naked eye. Some of the light propagating transversely through the fiber experiences guiding within the individual layers resulting in additional weak modes seen at photon energies between 1.7–2.4 eV captured in simulations and experiments. These modes only occur for light polarized in the fiber's axial direction (see Figure S3 in the Supporting Information). A detailed investigation of this effect is beyond the scope of this paper and will be addressed elsewhere.

The glass fiber that acts as the substrate for the multilayers in the rolling process can be removed from the fiber by dissolution in hydrofluoric acid or by simply pulling it out of the multilayer cladding. Once the glass core is removed, the fiber, being composed of two elastomers, can be elastically deformed by stretching it along its axis. An elongation along the fiber axis leads to a compression perpendicular to it, causing a decrease of its overall diameter and a reduction of the thickness of each individual layer. Due to the comparable Poisson's ratio of the constituent elastic materials the thickness ratio and the reflection intensity remain constant while the reflection band blue-shifts. This way, the reflected and transmitted color can be reversibly tuned by axial extension of the fibers (**Figure** **4**a,d). A reversible peak wavelength shift of over 200 nm has been recorded for axial elongations of a fiber to over 200% of its original length (Figure 4b,c).

**Figure 4:**
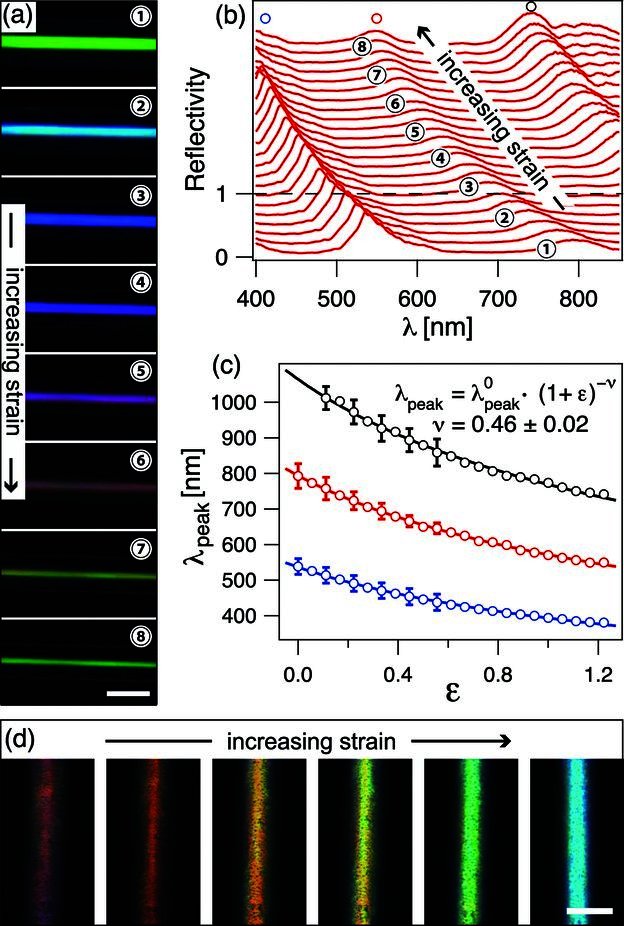
Mechanical tuning of the fibers' optical performance: (a) Optical micrographs of a fully elastic fiber showing the color tuning upon mechanical deformation induced by elongation along the fiber axis. The stiff glass core was removed from the fiber by pullout. Scale bar 50 μm. (b) The corresponding variation in the reflection spectrum. The bottom spectrum is shown on absolute scale (this particular fiber showed a peak reflectivity of over 70%) and subsequent spectra are offset from the previous one by 0.15. The numbers correspond to the numbered micrographs in (a). (c) Variation of peak wavelength *λ*_peak_ with applied strain *ε*. The open circles of different color correspond to three observed reflection peaks (labeled in (b) with corresponding open circles). The lines represent fits based on a power law that results from considering the fiber to be isotropically elastic, with the fit parameter *ν* representing the fiber's Poisson's ratio. The error bars shown for a selection of data points correspond to the standard deviation of the peak positions obtained for five consecutive stretch runs at different positions along the fiber. (d) Color tuning of a second fiber with different layer thickness where the glass core was removed by a hydrofluoric acid etch. The fiber color can be tuned throughout the whole visible spectrum. The dark spots on the fiber result from scattering caused by etching artifacts found in at least the top PDMS film in the multilayer cladding, which is also affected by the hydrofluoric acid. Scale bar 50 μm.

From Poisson's ratio and the proportionality between the thickness of individual layers in the cladding and the spectral band-gap position, the reflection peak center wavelength *λ*_peak_ is predicted to follow the relation λ_peak_ = λ^0^_peak_(1 + ε) ^−*v*^, where λ^0^_peak_ represents the reflection peak centre wavelength at zero axial strain, ε the applied axial strain and *v* the fibre's Poisson's ratio. Fitting the experimental data with this relation yields a Poisson's ratio of *v* = 0.46 ± 0.02 for the stretchable multilayer fibers matching the Poisson's ratios of the constituent rubber materials (Figure 4c). This is in good agreement with results obtained earlier for the deformation of planar elastic multilayers.^34^

In this paper, we have given an example of the versatility of bio-inspired approaches for the manufacture of novel photonic elements. The hierarchical photonic architecture in individual cells of the fruits of the tropical plant *Margaritaria nobilis* served as a model for the creation of novel tunable band-gap multilayer fibers with a large tuning range in the visible spectrum. Our room temperature fiber rolling technique allows fabrication of multilayer fibers with hundreds of layers from a wide range of polymeric material combinations that would not be realizable by conventional thermal fiber drawing. The fibers' band-gap center frequency can initially be tuned by adjusting the individual film thicknesses of the two constituent layers prior to the rolling process, which also allows shifting of the fibers' tuning range into the near UV or near IR. Chirped multilayer fibers can be realized by applying an appropriate force on the elastic bilayer during rolling of the multilayer cladding (see Figure S1 in the supp. inf.).

The incorporation of gold or silver layers into the concentric multilayer offers potential for the development of novel micron-scale fiber-based meta-materials. Asymmetric structures or chirality could be incorporated into the fibers by patterning of the initial bilayer prior to the rolling, promising additional interesting optical properties.^40^ Removal of the fiber core from inside the multilayer cladding permits mechanical deformation of fibers to more than twice their original length, which causes a tuning of the band-gap and a spectral blue-shift of over 200 nm. In the future, the incorporation of flexible core fibers will render the removal step obsolete. The flexibility in the choice of constituent materials for the multilayer fibers and their unique combination of mechanical and optical properties holds great potential for applications in mechanically tunable light guides or optical strain sensing. The fibers' mechanical flexibility and elasticity, in addition to the demonstrated color brilliance and tunability, can make them a versatile novel material for smart, color-dynamic textiles. The reported multilayer fiber manufacturing process can in principle be applied to a wide range of synthetic materials with varying optical and mechanical properties. Large area deposition of the initial bilayer can be achieved by spraying or blade coating in a roll-to-roll process before final rolling of the multilayer fibers. Hollow photonic fibers providing good thermal insulation can be produced by employing hollow polymeric micro-tubing as the inner fiber core. Exposure of the fibers to different solvents in the vapor or liquid phase would result in varying degrees of swelling and a corresponding reflection peak red-shift endowing the fibers or textiles made thereof with optical solvent sensing capacities. The final formation of the multilayer rolls could in principle also be achieved by self-induced rolling of the bilayer caused by directional stresses, which can be induced by gradually swelling one of the bilayer phases selectively.^41^

## Experimental Section

*Fiber Manufacture*: A thin polydimethylsiloxane (PDMS, Sylgard 184, Dow Corning) film was spun from a 4%wt solution in heptane onto a sacrificial water-soluble polystyrene-sulfonic acid layer on a silicon wafer. The PDMS film was cross-linked by curing it on a hotplate for 2h at 70 °C. Subsequently, a bilayer was formed by spin-coating a polystyrene-polyisoprene-polystyrene triblock copolymer (PSPI, Sigma Aldrich, 14%wt content polystyrene) film on top of the cross-linked PDMS layer from a 4%wt solution in toluene (Figure 1f left). Stripes of 12 cm by 1-2 cm of the bilayer were then released from the wafer onto the surface of a water bath. This was achieved by immersing the sample slowly into the water at an angle varying between 30–45° allowing the water to dissolve the sacrificial water-soluble film between the elastomer bilayer and the substrate, thereby detaching the bilayer from the substrate. A thin glass fiber (10-20 μm diameter) was then lowered onto the end of the floating bilayer, where it adhered to the PSPI film, the top layer in the bilayer. Once the core glass fiber had attached it was rotated at a speed of 10–20 turns per minute rolling up the bilayer to form the multilayer cladding (Figure 1f right).^34^, ^35^

*Structural and Optical Analysis*: Images of the cross-sections of the fruit's seed coat tissue cells were obtained by scanning electron microscopy (Hitachi S-3200N SEM). For imaging, the samples were coated with a 3 nm thick film of a gold/palladium alloy. The cells internal periodic structure was visualized by transmission electron microscopy (JEOL 100S TEM). Images were acquired after fixing and staining samples according to the protocol described elsewhere.^42^ Cross-sections of the fibers were obtained by cryo-fracture. Fractured fibers were coated with a 3-5nm thick platinum film to avoid charging artifacts during imaging and visualized using a field emission scanning electron microscope (Zeiss Supra55VP).

Simultaneous imaging and micro-spectroscopic spatial reflection/transmission intensity mapping of the fruit's surface and the fibers was performed in a modified optical microscope (Leica DMRX). The samples were illuminated in the area of interest with a halogen lamp in reflection or transmission. Via an additional microscope port, a fraction of the reflected light was collected confocally and guided by a fiber to a spectrometer (Maya 2000 Pro, Ocean Optics). The detection spot size depends on the diameter of the fiber and the magnification of the objective lens. Measurements with a 50x objective (NA = 0.55) and a fiber with 50 μm core diameter resulted in a spatial resolution of 1 μm. All spectra are referenced against a flat silver mirror of ≥95% reflectance in the wavelength range of 400–800 nm. In order to acquire spatially and spectrally resolved intensity distributions of a specific area on a sample, the sample was translated step-wise in the focal plane of the microscope with a minimum step size of 1μm using an automated, remote-controlled stage (Prior ES110). Individual spectra were acquired after each scanning step resulting in a complete map of the spectrally-resolved intensity distribution of the samples in reflection or transmission.

*Optical Modeling*: The transverse reflection and transmission of cylindrical multilayer fibers was modeled using the MIT Electromagnetic Equation Propagation package (MEEP)^36^ interfaced with custom-made C++ code to feed MEEP with the refractive index distributions in the simulation cell and to deduce the dispersion relations from the simulated field distributions. The fiber was modeled in cross-section with the layer thicknesses and the core fiber diameter acquired from the scanning electron microscope images of fiber cross-sections (d_PDMS_ = 240 nm, d_PSPI_ = 100 nm, d_core_ = 14 μm, Figure 1h,i) and refractive indices of the constituent materials measured by ellipsometry (n_PDMS_ = 1.41 n_PSPI_ = 1.54, n_core_ = 1.5). The modeling cell was bordered by absorbing boundaries simulating the effect of a finite numerical aperture of the microscope objective. A light pulse containing light of wavelengths 300–900 nm was propagated through the simulation cell. Detectors positioned above and below the fiber captured the electromagnetic fields as a function of time and space. The distance of the detectors to the fiber was chosen in order to detect waves propagating in an angular range that is commensurate with the numerical aperture of the microscope objective in the experiments. The detected fields served to deduce the reflection and transmission dispersion diagrams of the fibers using a suitable Fourier transformation algorithm to perform the far-field transformation and the spectral analysis. The simulations were repeated with periodic boundary conditions to ensure that no artifact spatial frequencies result from the absorbing boundaries and to ensure that conservation of energy was observed in the simulations (i.e reflection and transmission add up to unity).

## Supplementary material

As a service to our authors and readers, this journal provides supporting information supplied by the authors. Such materials are peer reviewed and may be re-organized for online delivery, but are not copy-edited or typeset. Technical support issues arising from supporting information (other than missing files) should be addressed to the authors.

## References

[1] Lee L, Szema R (2005). Science.

[2] Parker A, Townley H (2007). Nat. Nanotechnol.

[3] Biró LP, Vigneron JP (2011). Laser Photon. Rev.

[4] Vigneron JP, Rassart M, Vandenbem C, Lousse V, Deparis O, Biró LP, Dedouaire D, Cornet A, Defrance P (2006). Phys. Rev. E.

[5] Potyrailo R, Ghiradella H, Vertiatchikh A, Dovidenko K, Cournoyer JR, Olson E (2007). Nat. Photon.

[6] Hallam BT, Hiorns AG, Vukusic P (2009). Appl. Opt.

[7] Kolle M, Salgard-Cunha PM, Scherer MRJ, Huang F, Vukusic P, Mahajan S, Baumberg JJ, Steiner U (2010). Nat. Nanotechnol.

[8] Pris AD, Utturkar Y, Surman C, Morris WG, Vert A, Zalyubovskiy S, Deng T, Ghiradella HT, Potyrailo RA (2012). Nat. Photon.

[9] Chung K, Yu S, Heo C-J, Shim JW, Yang S-M, Han MG, Lee H-S, Jin Y, Lee SY, Park N, Shin JH (2012). Adv. Mater.

[10] Land MF (1972). Prog. Biophys. Mol. Bio.

[11] Kinoshita S, Yoshioka S, Miyazaki J (2008). Rep. Prog. Phys.

[12] Prum RO, Torres R (2003). J. Exp. Biol.

[13] Land MF, Nilsson DE (2001). Animal Eyes.

[14] Yoshida A, Motoyama M, Kosaku A, Miyamoto K (1997). Zool. Sci.

[15] Vukusic P, Hallam B, Noyes J (2007). Science.

[16] Vukusic P, Sambles JR, Lawrence CR (2004). Proc. R. Soc. London, Ser. B.

[17] Parker AR (2000). J. Opt. A: Pure Appl. Opt.

[18] Vukusic P, Sambles JR (2003). Nature.

[19] Kinoshita S, Yoshioka S (2005). ChemPhysChem.

[20] Vukusic P, Wootton RJ, Sambles JR (2004). Proc. R. Soc. London, Ser. B.

[21] Lee DW, Lowry JB (1975). Nature.

[22] Lee DW (1997). Am. Sci.

[23] Thomas KR, Kolle M, Whitney HM, Glover BJ, Steiner U (2010). J. R. Soc., Interface.

[24] Vignolini S, Rudall PJ, Rowland AV, Reed A, Moyroud E, Faden RB, Baumberg JJ, Glover BJ, Steiner U (2012). Proc. Natl. Acad. Sci. U. S. A.

[25] Galetti M, Levey DJ, Silva WR, Galetti M (2002). Seed Dispersal and Frugivory: Ecology, Evolution, and Conservation.

[26] Cazetta E, Zumstein LS, Melo-Júnior TA, Galetti M (2008). Rev. Bras. Bot.

[27] Aizenberg J, Weaver JC, Thanawala MS, Sundar VC, Morse DE, Fratzl P (2005). Science.

[28] Yeh P, Yarif A (1978). J. Opt. Soc. Am.

[29] Hart S, Maskaly GR, Temelkuran B, Prideaux PH, Joannopoulos JD, Fink Y (2002). Science.

[30] Temelkuran B, Hart S, Benoit G, Joannopoulos J, Fink Y (2002). Nature.

[31] Knight J (2003). Nature.

[32] Gauvreau B, Guo N, Schicker K, Stoeffler K, Boismenu F, Ajji A, Wingfield R, Dubois C, Skorobogatiy M (2008). Opt. Express.

[33] Abouraddy AF, Bayindir M, Benoit G, Hart SD, Kuriki K, Orf N, Shapira O, Sorin F, Temelkuran B, Fink Y (2007). Nat. Mater.

[34] Kolle M, Zheng B, Gibbons N, Baumberg JJ, Steiner U (2010). Opt. Express.

[35] Gibbons N, Baumberg JJ, Bower C, Kolle M, Steiner U (2009). Adv. Mater.

[36] Oskooi AF, Roundy D, Ibanescu M, Bermel P, Joannopoulos JD, Johnson SG (2010). Comput. Phys. Commun.

[37] Kreysing M, Pusch R, Haverkate D, Landsberger M, Engelmann J, Gentsch J, Mora-Ferrer C, Ulbricht E, Grosche J, Franze K, Streif S, Schumacher S, Makarov F, Guck J, Wolburg H, Bowmaker J, von der Emde G, Schuster S, Wagner H-J, Reichenbach A, Francke M (2012). Science.

[38] Born M, Wolf E (2005). Principles of Optics.

[39] Goodman JW (2005). Introduction to Fourier Optics.

[40] Huang FM, Sinha JK, Gibbons N, Bartlett PN, Baumberg JJ (2012). Appl. Phys. Lett.

[41] Luchnikov V, lonov L, Stamm M (2011). Macromol. Rapid Commun.

[42] Vukusic P, Kelly R, Hooper I (2008). J. R. Soc., Interface.

