# Supplementary material for: Bio-Inspired Band-Gap Tunable Elastic Optical Multilayer Fibers
Source: Adv Mater. 2013 Jan 27;25(15):2239–45. doi: 10.1002/adma.201203529 (PMC3652040; doi:10.1002/adma.201203529)
Supplement: Supplementary file 1 [file adma0025-2239-SD1.pdf]

Copyright WILEY-VCH Verlag GmbH & Co. KGaA, 69469 Weinheim, Germany, 2013.

ADVANCED MATERIALS

Supporting Information

for *Adv. Mater.*, DOI: 10.1002/adma.201203529

Bio-Inspired Band-Gap Tunable Elastic Optical Multilayer Fibers

Mathias Kolle, Alfred Lethbridge, Moritz Kreysing, Jeremy J. Baumberg, Joanna Aizenberg, and Peter Vukusic**

Supporting Information:

Variations in fiber geometry

Small modifications in the process of rolling the multilayer cladding onto the core fiber permit the realization of different internal geometries in the fiber. Attachment of the core fiber at the end of a free floating bilayer results in a fiber with one periodicity throughout the whole cladding (Fig. S1a). By attaching the core fiber a certain offset distance away from the edge of the bilayer film, claddings with two distinct multilayer periodicities can be realized (Fig. S1b). In this case, the inner section of the multilayer cladding has twice the periodicity of the outer section and the ratio of layer numbers in the inner and outer section can be controlled by controlling the distance between bilayer edge and fiber attachment line. Chirped multilayer claddings with a gradient in periodicity are realized by fixing the distance between the core fiber and the far edge of the bilayer during rolling leading to a successive stretching of the bilayer and a corresponding thinning of the rolled-up layers. As expected, such chirped multilayer fibers have a silvery appearance due to a wide reflection band.

Extraction of band-diagrams from experimental data

The band diagrams presented in the article were acquired from a fiber with two distinct periodicities in the cladding, the periodicity of the inner layers being about twice the periodicity of the outer layers which amounted to (350 ± 20) nm. The number of layers with double periodicity in the inner cladding zone is determined by the distance of the attachment line of the core fiber from the edge of the initial bilayer prior to the rolling (see Fig. S1b). The reflection and transmission data collected from the fiber in spatially resolved reflection and transmission intensity mappings (Fig.S2a) serve as the starting point for the extraction of the fiber's band-diagrams. The in-plane wave-vector k_x and the incidence angle θ of the incident light collected after reflection or transmission are directly related to the ratio $\bar{x} = {}^{x}/_{r}$ between

the fractional lateral distance x of the emerging ray from the axis of the fiber and its radius r (Fig. S2b). Taking into account the fiber geometry and the directionality of light detection this in-plane wave vector component k_x^R of the incident light collected after reflection is defined by $k_x^R = k_0 \sin(\theta_\mathrm{R}) = k_0 \sin(2sin^{-1}(\bar{x}))$, where $k_0 = {}^{2\pi}/_{\lambda}$, λ is the wavelength and θ_R is the incidence angle of the reflected light (Fig. S2b, top). The in-plane wave vector component k_x^T of incident light transmitted transversely through the fibre (Fig. S2b, bottom) is defined by $k_x^R = k_0 \sin(\theta_\mathrm{R}) = k_0 \sin(2\,(sin^{-1}(\bar{x}) - \sin^{-1}({}^{\bar{x}}/_{n_\mathrm{eff}})))$, where θ_T is the incidence angle of the collected transmitted light and n_eff is the effective refractive index of the fiber, approximately given by $n_\mathrm{eff} \approx \sqrt{\frac{n_1^2 d_1 + n_2^2 d_2}{d_1 + d_2}}$ with $n_{1,2}$, $d_{1,2}$ being the refractive indices and thicknesses of the constituent layers.

The resulting dispersion relations are plotted in Fig. S2c against the in-plane wave vector k_x of the incident light. To account for the limited numerical aperture of the illumination and collection optics in the micro-spectroscope the band diagrams have to be corrected by the optical transfer function (OTF) of the setup. The OTF is given by the overlap of numerical aperture of the collection optics and the light cone emerging from the fiber surface at an angle θ after reflection or transmission (Fig. S2d).[1] This is schematically represented in Fig. S2d for reflection (top) and transmission (bottom). The overlap area defining the OTF is shown in green. The numerical apertures in these schematics are not drawn to scale. In the reflection measurements the illumination and collection is achieved via the same 50x objective with a numerical aperture of 0.55 resulting in the optical transfer function plotted in Fig. S2d (blue line) against the angle θ between incident and emerging light cone, which is determined by the orientation of the fibre surface at the collection point. In the transmission measurements a condenser with a numerical aperture of 0.9 was used. The resulting band-diagrams after correction for the OTF of the setup are shown in Fig. S2e).

Guided modes in the fiber cladding

Finite Difference Time Domain simulations employing the freely available software package MEEP[2] were carried out to identify the origin of an additional mode found in the transmission band-diagram in the range around 2eV to 2.5eV. Simulations of the reflection and transmission of a fiber with single periodicity show that this mode only exists for perpendicularly polarized light, i.e. light with its electric field vector aligned with the fiber axis (Fig. S3a). Monitoring the total energy in the simulation cell during the length of the simulation reveals the differences between the two orthogonal polarizations (Fig. S3b). The features in the decay of energy over time for both polarizations in the simulation cell can clearly be matched to specific events during the simulation visualized by representing field distributions (on log scale) at given times (Fig. S3c, numbers in the images correspond to numbers in (b)). Abrupt decays of total energy in the cell early on in the simulation relate to the exit of reflected waves at the top boundary (Fig. S3c 1,2) and scattering of waves that subsequently leave at the side walls (Fig. S3b 4,5). Significant differences between the two polarization states are the manifestation of the Brewster effect for parallel-polarized light (Fig. S3c 1, bottom) and the proportionally stronger scattering of perpendicularly polarized light (Fig. S3c 3,4). As a consequence, proportionally more parallel-polarized light is transmitted through the fiber. The most significant decay in total energy in the simulation cell results from the exit of waves after transmission through the fiber at the lower simulation cell boundary (Fig. S3c 5,6). That decay is proportionally more pronounced for the parallel-polarized light component. This difference resulting from the Brewster effect sets the differences in the transmission distributions of the two light polarizations at higher in-plane wave vector component k_x. After exit of the majority of light in transmission from both polarisation states a significantly higher total energy remains in the cell for perpendicularly polarized light resulting from the coupling of waves into guided modes in the fiber cladding (compare (Fig.

S3c 5,6 top with 5,6 bottom). This guiding in the cladding, which is more effective in confining light with perpendicular field vector is the origin for the mode that is observed only in transmission.

[1] J.W. Goodman, *Introduction to Fourier Optics*, Roberts and Company Publishers, Greenwood Village, USA, **2005**.

[2] A. F. Oskooi, D. Roundy, M. Ibanescu, P. Bermel, J. D. Joannopoulos, S. G. Johnson, *Computer Physics Communications* **2010**, *181*, 687.

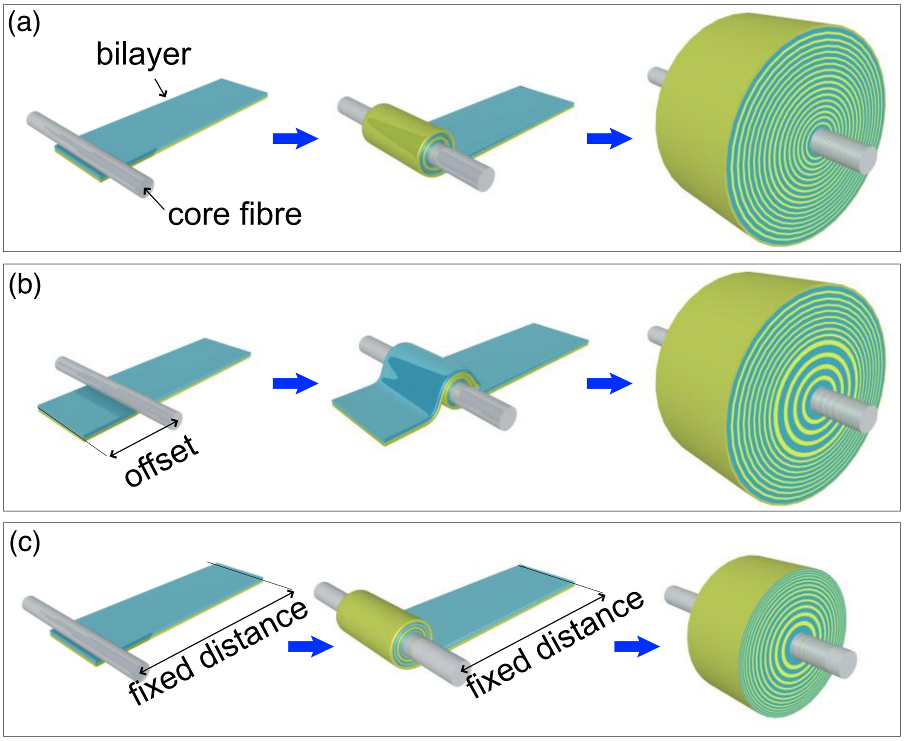

Figure S1: Fiber-internal geometries. A set of simple internal geometries can be achieved by small modifications of the manufacturing procedure. (a) Rolling of fibres with a single periodicity. (b) Realization of fibers with two internal periodicities. (c) Rolling of fibers with gradual thickness variation.

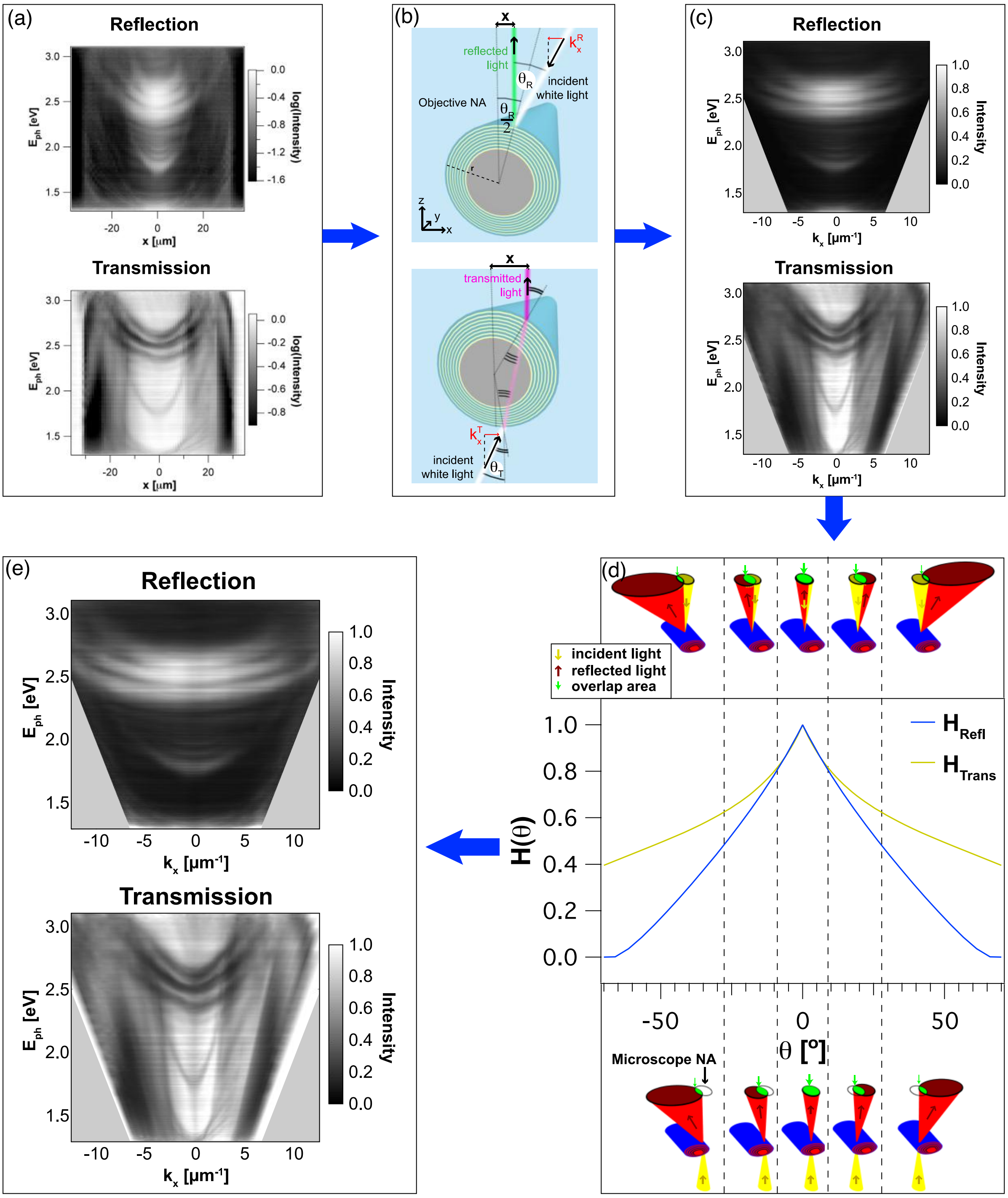

Figure S2: Deduction of band diagrams from radial intensity distributions of fiber transmission and reflection. (a) The original data acquired by micro-spectroscopy showing the radial intensity distribution of fiber reflection and transmission. (b) Schematic representation of the geometrical relations between measured radial projected distance *x* and light incidence angle $\theta_{\mathrm{R,T}}$ for reflection and transmission. (c) Data deduced by taking into account the relation between x and $\theta_{\mathrm{R,T}}$. Optical transfer function of the microscope $H(\theta_{\mathrm{R,T}})$ for measurements in transmission (yellow) and reflection (blue). Simplified schematic illustrations of the origin of the setup's optical transfer function as the overlap (green intersection) between reflected light (top row) or transmitted (bottom row) light. The numerical aperture of condenser and objective are not drawn to scale (d) Reflection and transmission band-diagrams after all corrections are applied.

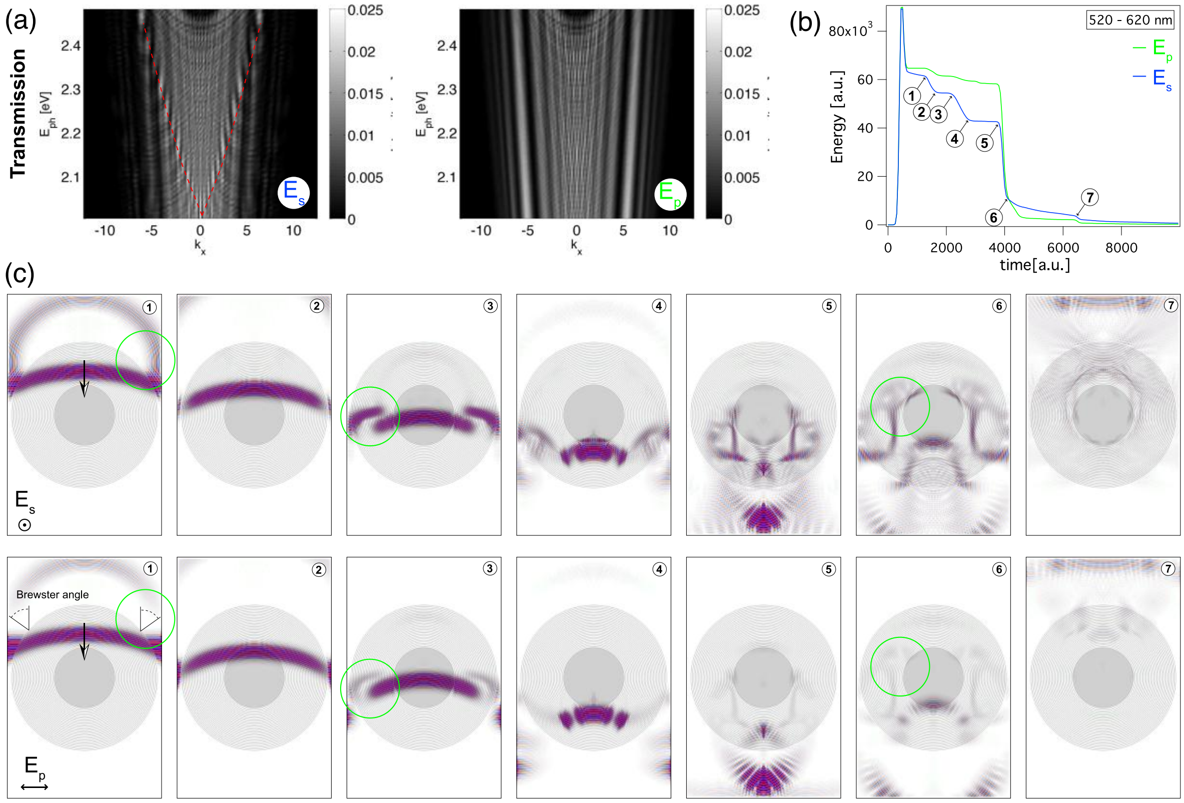

Figure S3: Polarization dependent guiding of light in the fiber cladding. (a) Simulated transmission band-diagrams for perpendicular (E_{s}) and parallel (E_{p}) polarised light in the range of 2eV - 2.5eV for a fibre of 340nm cladding periodicity. The red dashed light in the transmission band-diagram underlines the presence of a guided mode for perpendicularly polarized light. (b) Total energy decay in the simulation cell as a function of simulation time for parallel and perpendicularly polarized light. The numbers mark different pathways of energy leaving the cell. (1)(2) - reflected light leaving at the upper boundary, (3)(4) - scattered waves leaving the cell at the sidewalls, (5)(6) transmitted waves exiting at the lower boundary, (7) - waves leaving at the upper boundary after incurring internal reflection within the fiber. (c) Color-coded representation of the logarithm of the field distribution of perpendicularly (top) and parallel (bottom) polarized light for the time points enumerated in (b). Green circles point out the differences between the two polarization states.
